# The centrosome–Golgi apparatus nexus

**DOI:** 10.1098/rstb.2013.0462

**Published:** 2014-09-05

**Authors:** Rosa M. Rios

**Affiliations:** Cell Signalling Department, CABIMER-CSIC, Seville 41092, Spain

**Keywords:** centrosome, Golgi apparatus, microtubules, AKAP450

## Abstract

A shared feature among all microtubule (MT)-dependent processes is the requirement for MTs to be organized in arrays of defined geometry. At a fundamental level, this is achieved by precisely controlling the timing and localization of the nucleation events that give rise to new MTs. To this end, MT nucleation is restricted to specific subcellular sites called MT-organizing centres. The primary MT-organizing centre in proliferating animal cells is the centrosome. However, the discovery of MT nucleation capacity of the Golgi apparatus (GA) has substantially changed our understanding of MT network organization in interphase cells. Interestingly, MT nucleation at the Golgi apparently relies on multiprotein complexes, similar to those present at the centrosome, that assemble at the *cis*-face of the organelle. In this process, AKAP450 plays a central role, acting as a scaffold to recruit other centrosomal proteins important for MT generation. MT arrays derived from either the centrosome or the GA differ in their geometry, probably reflecting their different, yet complementary, functions. Here, I review our current understanding of the molecular mechanisms involved in MT nucleation at the GA and how Golgi- and centrosome-based MT arrays work in concert to ensure the formation of a pericentrosomal polarized continuous Golgi ribbon structure, a critical feature for cell polarity in mammalian cells. In addition, I comment on the important role of the Golgi-nucleated MTs in organizing specialized MT arrays that serve specific functions in terminally differentiated cells.

## 1. Introduction

The microtubule (MT) cytoskeleton is critically important for the organization of eukaryotic cells and plays a central role in the regulation of a wide variety of cellular processes. The organization and nucleation of MTs must be highly regulated in order to generate and maintain MT complex arrays. In most model systems studied so far, MT nucleation relies on γ-tubulin complexes that control MT formation spatio-temporally [1]. γ-Tubulin complexes are necessary because spontaneous nucleation of new tubulin polymers is kinetically limiting both *in vivo* and *in vitro*. Surprisingly, the majority of γ-tubulin-containing complexes are found in the cytoplasm, where they are devoid of significant MT nucleation activity [2]. This raises the question as to how γ-tubulin nucleating complexes are recruited and then activated at specific intracellular locations and how this recruitment is regulated. An answer to this question is found in the activity of MT-organizing centres.

The major MT-organizing centre in animal cells is the centrosome that consists of a pair of centrioles surrounded by a pericentriolar matrix (PCM) [3]. It orchestrates MT organization by stimulating MT nucleation and anchoring. These activities mostly reside on the PCM which is highly enriched in γ-tubulin nucleating complexes. Several PCM components are known to serve for γ-tubulin recruitment, and their roles in MT nucleation during the cell cycle have been widely studied [4]. Interestingly, some of these centrosomal proteins also localize at the Golgi apparatus (GA), which has been shown to act as an important MT-nucleating centre [5]. In this review, I will focus on the roles of PCM proteins at the GA as compared to those they perform at the centrosome. I will briefly introduce our current knowledge about MT nucleation at the centrosome. Then I will analyse the mechanisms and functions of MT nucleation at the GA and its regulation during mitosis. Finally, I will provide an overview on recent advances on our understanding of the potential mechanisms by which the GA contributes to generate specialized MT arrays in differentiated cells.

## 2. Microtubule nucleation at the centrosome

### (a) Overview of pericentriolar matrix organization

The PCM consists of a meshwork of fibrous proteins [3]. Both its size and ability to nucleate MTs are tightly regulated during the cell cycle and during cell differentiation. As cells enter mitosis, the PCM undergoes a drastic increase in size. This process, referred to as centrosome maturation, occurs during the G2/prophase transition and is driven by the accumulation and activation of γ-tubulin and other PCM proteins [6]. In contrast, the centrosome loses its function as an MT-organizing centre during differentiation of some tissues such as epithelia, muscles and neurons. In these cases, the amount of γ-tubulin at the centrosome decreases and the majority of MTs arise from acentrosomal sites [7,8].

The PCM has been traditionally considered an amorphous structure, probably due to its rather homogeneous density in electron microscopy images. Recently, the application of subdiffraction-resolution fluorescence microscopy combined with site-specific antibody analyses has unveiled a high-order spatial organization of the PCM [9–12]. During interphase, the PCM is arranged as concentric layers with distinct molecular composition and architecture. Some PCM components adopt ring-like distributions located at specific distances from the centriole walls, whereas other PCM components show an elongated orientation and extend radially from the centriole wall towards the periphery, thus spanning several layers. During mitosis, this concentric organization is less defined and PCM proteins appear organized as extended networks [10–12]. To elucidate how this highly ordered organization contributes to the primary PCM functional role, which is to nucleate and anchor MTs, is a major task for future studies. It would also be interesting to evaluate the significance of the PCM architecture in generating radial arrays of MTs.

### (b) Mechanisms of microtubule nucleation

Centrosomal MT nucleation is mediated by a large protein complex named the γ-tubulin ring complex (γ-TuRC) due to its striking ring shape in electron micrographs [1]. In addition to γ-tubulin, the γ-TuRC contains five homologous γ-tubulin complex proteins (GCPs; GCP2 to GCP6). The conserved essential core of the MT nucleating machinery is the γ-TuSC that consists of two copies of γ-tubulin bound to GCP2 and GCP3. Multiple copies of the γ-TuSC associate with GCP4, GCP5 and GCP6 and this association contributes to formation of the characteristic structure of the γ-TuRC. Several other proteins including MOZART1, MOZART2 (or GCP8) and NEDD1 (also called GCP-WD or GCP7) have also been described as components of the human γ-TuRC but they might have a regulatory rather than a structural role [13–15].

Although recent structural work has shed light on the mechanism of γ-TuRC-based MT nucleation [16], the molecular details of γ-TuRC recruitment to the centrosome are still not completely understood. Centrosomal attachment seems to occur through interaction with γ-TuSC components since in *Saccharomyces cerevisiae,* naturally lacking GCP4 and GCP6 proteins, γ-TuSC components are still found at the spindle-pole body, the functional analogue of the centrosome. And in other organisms, although present, GCP4 to GCP6 are dispensable for γ-TuSC centrosomal localization [1,4]. However, in humans centrosomal targeting of γ-tubulin requires an intact γ-TuRC [15,17]. Recruitment of γ-TuRC to centrosomes at different cell cycle stages involves several centrosomal proteins. Among them, Cep192 was shown to be required for the recruitment of NEDD1, one of major γ-tubulin recruiting factors [17,18]. Cep192 and NEDD1 silencing resulted in the loss of functional centrosomes in mitotic but not interphasic cells, suggesting that they are involved in the centrosome maturation process and in bipolar spindle assembly [19,20]. On the contrary, ninein-like protein recruits γ-TuRCs to the centrosome and stimulates MT nucleation specifically during interphase [21]. At the onset of mitosis, these three proteins (Cep192, NEDD1 and ninein-like protein) are phosphorylated by PLK1, the main protein kinase responsible for centrosome maturation at G2/M transition. However, while PLK1 activation and subsequent phosphorylation of Cep192 and NEDD1 result in their accumulation at the centrosome, PLK1-mediated phosphorylation of ninein-like protein triggers its displacement from the centrosome and inhibits its dynein–dynactin-dependent intracellular transport towards the centrosome [19–22].

AKAP450 (also known as AKAP350 or CG-NAP) and both pericentrin isoforms (A and B, also known as kendrin [23]) interact with GCP2/GCP3 components of γ-TuRCs [24,25]. These two proteins share a common C-terminal domain called PACT domain that targets them at the centrosome, whereas the N-terminal domain mediates their binding to GCP2/GCP3 [25,26]. Finally, CDK5Rap2 directly binds to GCP4 through a motif called γ-TuNA that has been described as a strong activator of MT nucleation [27,28]. The γ-TuNA motif is located at the conserved N-terminal CNN1 domain and is also present in myomegalin, a CDK5Rap2 paralogue [29]. Depletion of each of these proteins, their release from the centrosome or disruption of their interactions with γ-TuRCs leads to defects in MT nucleation either in interphase or in mitosis or in both [24,27,30,31].

Interestingly, several studies have reported mutual interactions among these proteins. Thus, AKAP450 interacts with pericentrin and both of these proteins bind to CDK5Rap2 [25,32,33]. These proteins are also interdependent for their localization at the centrosome. Pericentrin drives CDK5Rap2 recruitment to the centrosome [11,31,32]. CDK5Rap2 mediates AKAP450 centrosomal targeting [33] and AKAP450, in turn, recruits myomegalin [29]. Since these are all large structural proteins that form coiled-coil interactions, they all are putative scaffolding components of the PCM. These structural properties together with their ability to recruit γ-tubulin and their interdependence for centrosomal targeting point to the possibility that they form multiprotein complexes essential for both PCM organization and MT generation at different phases of the cell cycle. Further studies, including three-dimensional super-resolution microscopy, are required to define their contribution to PCM architecture as well as their precise function and regulation during the cell cycle.

### (c) Microtubule anchoring at the centrosome

In addition to MT nucleation, MT growth and dynamics require other centrosome-associated activities, most importantly MT capping and MT anchoring. A link between all these centrosome-dependent processes is found in the role carried out by ninein [34]. Ninein localizes at the subdistal appendages of mother centrioles that are thought to be a major site for MT anchoring. Ninein targets the centriole via its C-terminus and recruits γ-tubulin-containing complexes via its N-terminus [34]. However, it remains unknown whether ninein can anchor MTs directly or whether it acts in conjunction with other anchoring proteins that are also present at the subdistal appendages of the mother centriole such as the largest subunit of the dynactin complex p150Glued or the MT plus-end-associated protein EB1. Both ninein and ninein-like protein associate with dynactin–dynein complexes, suggesting that they can act in collaboration with p150Glued to carry out their anchoring function [22,34]. Furthermore, AKAP450 interacts with p150Glued and this interaction could provide additional MT-anchoring properties to the PCM [35,36]. Finally, CAP350 has also been shown to be involved in MT anchorage at the centrosome. CAP350 is a large non-coiled-coil and highly conserved CAP-Gly centrosomal protein that directly binds to MTs. In addition, CAP350 has been shown to recruit FOP to the centrosome and FOP, in turn, recruits EB1. Depletion of either CAP350, FOP or EB1 causes loss of MT anchoring and disorganization of the radial MT array in interphase cells [37].

**Figure 1.**
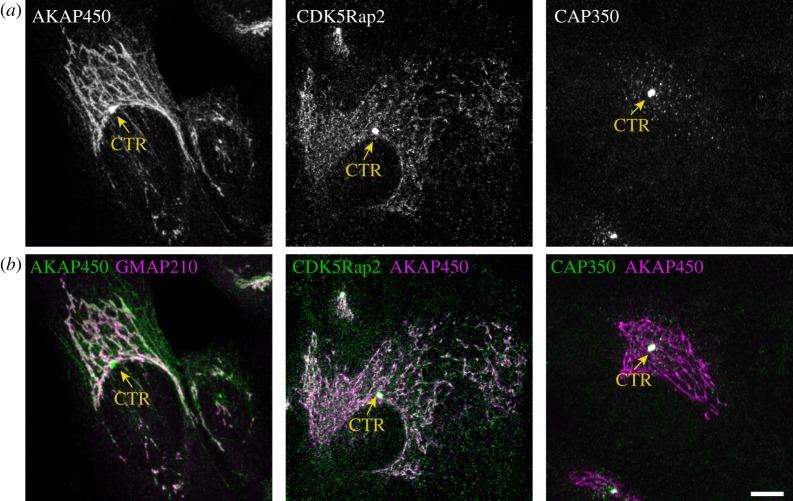
Subcellular localization of AKAP450, CDK5Rap2 and CAP350 by immunofluorescence analysis of interphasic RPE-1 epithelial cells (R. M. Rios 2010 & 2011, unpublished results). (*a*) Shows single labellings and (*b*) double immunofluorescence stainings of these proteins (as indicated). GMAP210 was included as a Golgi marker in the line at left. Arrows indicate the position of the centrosome. Scale bar, 10 μm.

So far, five of the PCM proteins described above (AKAP450 [38], CDK5Rap2 [39], myomegalin [40], CAP350 [41] and pericentrin [8]) have been found associated with the GA in mammalian cells (see also figure 1). Currently available data suggesting that they might play similar roles at both subcellular locations has largely modified our vision of the mechanisms governing not only Golgi organization but also complex MT array generation in differentiated cells.

## 3. Microtubule nucleation at the Golgi apparatus

### (a) The pericentrosomal Golgi apparatus in mammalian cells

The GA is the central organelle of the eukaryotic secretory pathway performing different functions essential for cell growth, homeostasis and division. Although its basic function is highly conserved, the GA varies greatly in shape from one organism to another. In the simplest organisms such as *S. cerevisiae*, this organelle assumes a unique form consisting of dispersed cisternae or of isolated tubular networks [42]. Unicellular green alga [43] and many protozoa organize the GA as a single pile of flattened cisternae aligned in parallel [44]. This organization of the GA is referred to as a Golgi stack and represents the basic structural unit of the GA. In fungi [45], plants [46] and *Drosophila* [47], many separate Golgi stacks are dispersed throughout the cytoplasm. Each Golgi stack is associated with a single endoplasmic reticulum exit site, forming a secretory unit. By contrast, in vertebrate cells individual Golgi stacks are laterally connected to form a continuous membrane system called the Golgi ribbon [48,49].

In most vertebrate cells, the Golgi ribbon localizes near the nucleus and surrounds the centrosome. The pericentrosomally positioned GA, in combination with oriented MT arrays, defines an axis of secretion that is relevant for many physiological processes. Time-lapse microscopy studies revealed that the overall three-dimensional arrangement of the GA as a pericentrosomal ribbon is quite stable in spite of intense membrane trafficking [50]. Both integrity and pericentrosomal positioning depend on MTs and dynein [51–54]. Dynein, which is recruited to the GA by the peripheral coiled-coil protein golgin 160 [55], moves Golgi elements from the cell periphery towards the cell centre. Once there, active anchoring or tethering to the centrosome might further maintain their pericentrosomal position. GMAP210 is a good candidate to carry out this activity, since when targeted to mitochondria it induces their clustering around the centrosome and when depleted it yields immotile, dispersed Golgi stacks [56,57]. GMAP210 is a peripheral homodimeric *cis*-Golgi protein that binds MT minus-ends and γ-tubulin [58]. GMAP210 has at least two membrane targeting motifs located at the ends of the protein, both of which are required to ensure its proper *cis*-Golgi localization and function [59,60]. These results reveal a role for GMAP210 in maintaining Golgi ribbon positioning and integrity, probably by participating in the formation of connecting *cis*-cisternae [57]. Dispersed Golgi stacks in GMAP210-depleted cells are competent for general protein transport to the cell surface excluding an essential role for GMAP210 in membrane trafficking [56].

Support for a role of GMAP210 in Golgi architecture came from the analysis of mutagenized mice dying from an autosomal recessive neonatal lethal skeletal dysplasia [61]. This dysplasia shares common phenotypic features with achondrogenesis type 1A in humans. Both affected mice and patients have nonsense mutations in the *Trip11* gene, which encodes GMAP210. Loss of GMAP210 altered the Golgi structure in many (but not all) tissues and compromised normal glycosylation in the Golgi as well as the transport of certain proteins that would normally be destined for the extracellular matrix [61]. Follit and colleagues, using an alternative approach, also engineered a GMAP210 deficient mouse. Embryonic kidney cells derived from this knockout mouse exhibited an apparently normal Golgi complex, although the structure of the GA in other tissues was not examined [62]. GMAP210 is also the receptor of IFT20 protein at the GA [62]. IFT20 is a critical component of the intraflagellar transport machinery required for the formation and extension of the primary cilium. In mouse embryonic kidney cells lacking GMAP210, primary cilia are shorter than normal and contain reduced amounts of the membrane protein polycystin-2, suggesting that GMAP210 and IFT20 function together at the Golgi in the sorting or the transport of proteins destined for the ciliary membrane [62]. Accordingly, the homologue of GMAP210 in *Caenorhabditis elegans* is involved in maintaining Golgi organization and in the regulation of cilium length [63].

### (b) Mechanisms of microtubule nucleation at the Golgi apparatus

In the past few years, a new concept about the role of the GA in MT dynamics has emerged: the GA acting as an MT-organizing centre. In a pioneering study, Christian Pous’s group in 2001 [64] reported that Golgi membranes were able to assemble and stabilize MTs in hepatic cells after nocodazole treatment. In addition, purified Golgi membranes were shown to contain γ-tubulin and to promote MT assembly, a finding that has recently been confirmed in neurons [64,65]. By tracking polymerizing MTs, Kaverina's group then unambiguously demonstrated that an MT subset grows directly from Golgi membranes [5]. Two other important findings from this study were that siRNA-mediated depletion of γ-tubulin inhibits both Golgi and centrosome MT generation, and that laser ablation of the centrosome does not affect the number of MTs formed at the GA. It was estimated that up to 50% of MTs in RPE-1 interphasic cells are produced by the GA. MT nucleation at the GA was shown to require the MT-stabilizing activity provided by CLASPs, MT plus-end binding proteins that are recruited to the GA through the interaction with the TGN-associated protein GCC185 [5].

This study did not identify, however, the machinery responsible for MT nucleation at the GA. We further demonstrated that MT nucleation at the GA depends on the PCM protein AKAP450 that specifically associates with the *cis*-GA [66]. Depletion of Golgi-associated AKAP450 or expression of a dominant negative mutant, which dissociated AKAP450 from the GA but not from the centrosome, completely abolished MT nucleation at the GA [36,66]. We then proposed that the *cis*-GA becomes a major site for MT nucleation by acting as a preferential γ-TuRC docking site through a mechanism similar to that operating at the centrosome. This hypothesis has received additional support with the finding that other γ-TuRC recruiting PCM proteins such as CDK5Rap2 (figure 1) and myomegalin localize at the *cis*-GA [29,39].

**Figure 2.**
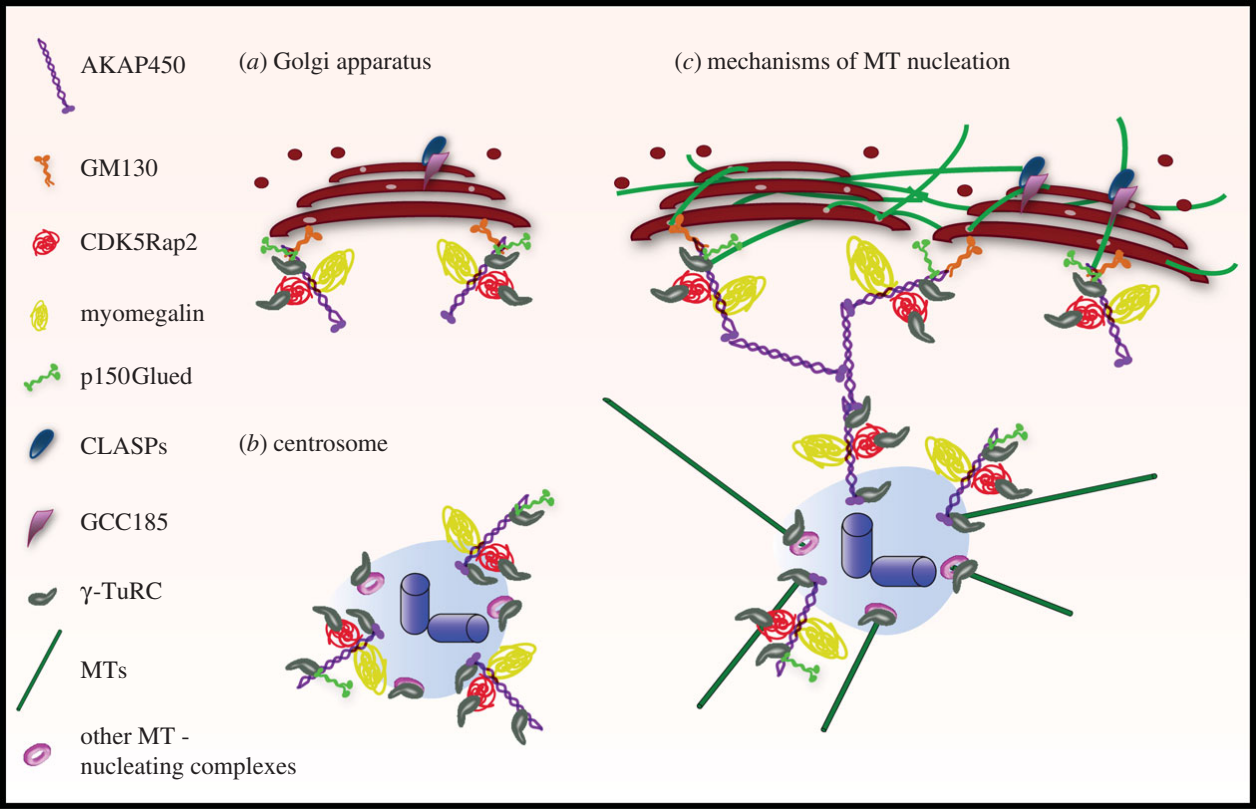
The centrosome–GA nexus. (*a,b*) Similar multiprotein complexes are present at both the *cis*-face of the GA (*a*) and the PCM (*b*). These complexes contain AKAP450, CDK5Rap2, myomegalin and MT-anchoring proteins such as p150Glued. They are specifically recruited to the *cis*-GA through the interaction between GM130 and the N-terminal domain of AKAP450. Targeting to the centrosome is mediated by the AKAP450 C-terminal PACT domain. AKAP450 and CDK5Rap2 recruit γ-TuRCs and promote MT nucleation at both subcellular locations. Myomegalin and p150Glued might provide MT stabilization activities. (*c*) A working model for the mechanism of MT nucleation at the GA based on availaible data. During Golgi assembly, an MT nucleated by one multiprotein complex might be stabilized by another one located in the vicinity of the same or of a neighbouring stack, before being captured by TGN-associated CLASPs. This would facilitate the correct alignment of Golgi stacks preceding their fusion into a ribbon. MTs could stop growing at the TGN, thus generating an intra-GA network. Alternatively, they may continue to elongate towards the cell periphery. I propose that proteins such as AKAP450 or CDK5Rap2 represent not only functional but also physical connectors between the centrosome and the GA in mammalian cells.

Indeed, recent data suggest that Golgi capacity to nucleate MTs might rely on multiprotein complexes similar to those present at the PCM (see figure 2 for a model). These data also point out a hierarchy in the association of these proteins to the Golgi. Thus, the peripheral *cis*-Golgi protein GM130 recruits AKAP450 [66] that, in turn, recruits both CDK5Rap2 and myomegalin [29,39]. Accordingly, GM130 depletion caused the dissociation of both AKAP450 and myomegalin from the GA [29], whereas brefeldin A treatment induced redistribution of GM130, AKAP450 and myomegalin to endoplasmic reticulum exit sites that concomitantly acquired MT nucleation capacity [29,66]. It is worth noting that although significant cytoplasmic pools of AKAP450, CDK5Rap2, myomegalin and γ-tubulin exist, MTs do not normally form in the cytoplasm. This suggests that multiprotein complexes become competent for MT nucleation only after being assembled at the *cis*-Golgi membrane surface. Interestingly, a cytoplasmic pool of GM130 is lacking, and GM130 is exclusively present at the *cis*-GA [57,66]. Thus, it appears as a critical factor for MT nucleation at the GA by controlling both localization and rate of the process.

Despite these data, the specific mechanism whereby AKAP450 induces MT formation at the GA has not been fully elucidated. AKAP450 could recruit γ-TuRC directly and/or indirectly through CDK5Rap2 (see model in figure 2). AKAP450, CDK5Rap2 and their respective orthologues in other species have been reported to bind γ-tubulin-containing complexes [25,27,67,68]. Takahashi *et al.* [25] showed that the N-terminal region of CG-NAP indirectly associates with γ-tubulin through interaction with GCP2/GCP3 components of γ-TuRC. We have not detected any interaction between γ-tubulin and the most N-terminal part of the protein in spite of careful examination [36], although the truncated mutants used in both studies were not identical, which could explain the discrepancies. However, CDK5Rap2 directly binds γ-TuRC and works as a strong activator of MT nucleation through its γ-TuNA motif [28].

Based on the capacity of AKAP450 to bind p150Glued and the finding that blocking dynein/dynactin interferes with GA-based MT nucleation at the GA [66], it has been proposed that AKAP450 might also support MT formation via a dynein/dynactin-dependent mechanism [69]. The binding site for p150Glued is localized at the N-terminus of AKAP450, close to the GM130-interacting motif. An AKAP450-truncated mutant containing both p150Glued and GM130-binding motifs targets the GA and MTs, and yet GA membranes are unable to nucleate MTs [36]. Therefore, although a direct proof is still lacking, all evidence supports the idea that the main mechanism for MT nucleation at the GA is based on γ-TuRC recruitment. Interestingly, the PTTG1/securin protein has been found in a complex with AKAP450, GM130 and γ-tubulin [70]. PTTG1/securin localizes at both the centrosome and the GA, and when depleted, MT nucleation is delayed at both subcellular localizations. Based on described PTTG1/securin functions, the authors proposed that PTTG1 could act as a chaperone contributing to the formation and stability of MT-nucleating complexes.

In addition to AKAP450-mediated MT nucleation, successful formation of MTs from the GA also requires the MT-plus-end stabilizing activity provided by CLASPs [5]. However, AKAP450 localizes at the *cis*-Golgi, whereas CLASPs, on the contrary, bind to the TGN membranes. How these proteins, localized at opposite faces of Golgi stacks, mechanistically cooperate to promote MT nucleation and growth is intriguing. Most cell types display a dense meshwork of short MTs that colocalize with the GA. This intra-Golgi MT network is lacking in cells depleted of either CLASPs or AKAP450 [5,66]. One straightforward possibility to explain how this network is assembled is that MTs elongate from the *cis*-face of the Golgi stacks and their plus-ends are capped by *trans*-Golgi network (TGN)-associated CLASPs. This would generate short MTs within, and rather parallel to, the Golgi ribbon. Interestingly, while centrosomal MTs show clear radial organization, MTs formed at the Golgi are predominantly tangential [69]. This geometry would favour tangential linking and fusion of Golgi stacks into a Golgi ribbon that is the primary function of Golgi-nucleated MTs (see §3*c*). Another subpopulation of Golgi-nucleated MTs is directed towards the front of motile cells [5]. In this case, CLASPs might stabilize *cis*-Golgi-nucleated MTs growing towards the TGN, thus allowing them to extend away towards the cell periphery.

It should also be taken into account that all the proteins forming part of putative MT-nucleating multiprotein complexes at the *cis*-Golgi might also contribute to MT anchoring and stabilization. As mentioned above, AKAP450 interacts with p150Glued that directly binds to MTs and EB1. CDK5Rap2 was shown to bind to growing MT tips by associating with EB1, suggesting that it could, in this way, regulate the plus-end dynamics of MTs [71]. Moreover, two myomegalin isoforms differing at their N-terminus have been identified in RPE-1 cells [29]. One of them contains the CNN1 domain also shared by CDK5Rap2 that confers upon them the capacity to bind γ-TuRC. This isoform is present at both the centrosome and the GA. The second isoform lacks this domain but it is able to bind EB1. Notably, this second isoform specifically associates with the GA. Although neither EB1 nor its relative EB3 have yet been observed at the GA, it is tempting to speculate that MT nucleation and EB1-mediated stabilization activities are present in the same complex, or in very close proximity, at the *cis*-Golgi. This might contribute to efficient and coordinated growth of MTs at the *cis*-GA before being stabilized at the TGN. Supporting this view, time-lapse imaging of nocodazole-recovering cells showed that tips of MTs growing from Golgi stacks were covered with GFP-EB3 from the very beginning of MT nucleation [5]. This model also agrees with early three-dimensional electron microscopy studies, in which individual MTs and their relationships with cisternae were analysed *in situ*. These studies revealed that MTs associate with the first *cis*-cisternae of the GA over long distances and also cross Golgi stacks at multiple points via non-compact regions and cisternal openings [72] (figure 2).

Finally, the centrosomal protein CAP350 has been shown to specifically stabilize Golgi-associated MTs in HeLa cells [41]. CAP350 not only localizes at the centrosome, but is also present as numerous dots in the Golgi area and as a significant pool in the cytoplasm. Unexpectedly, CAP350 was found to bind MTs through its N-terminal domain rather than through its CAP-Gly domain. When overexpressed, CAP350 targets the centrosome but also binds to MTs colocalizing with the GA. At higher expression levels, exogenous CAP350 covers the whole MT network. Perturbation of CAP350 expression levels results in Golgi fragmentation but not dispersal, suggesting that it participates in maintaining Golgi ribbon integrity.

### (c) Functions of Golgi-nucleated microtubules

In contrast to the effect on Golgi morphology and positioning induced by MT depolymerization or dynein-activity inhibition, blocking of MT nucleation at the GA does not result in Golgi dispersal. In partially CLASPs- or AKAP450-depleted cells, a circular GA surrounding the centrosome is observed [5,66]. FRAP experiments revealed, however, that the GA is highly fragmented, suggesting that Golgi elements are unable to form a continuous Golgi ribbon under these conditions [36,73]. These results suggested that centrosomal MTs might support central Golgi positioning but would be insufficient for proper Golgi ribbon formation and that, conversely, Golgi-based MTs might be dispensable for translocation of membrane elements from the cell periphery towards the cell centre but required for their fusion into a continuous structure. Experimental support for this hypothesis was provided by Kaverina and colleagues and further confirmed by us [36,73]. During nocodazole recovery, the subset of Golgi-nucleated MTs was first required for the assembly of Golgi fragments into larger elements at the cell periphery. Then, as a second step, centrosomal MTs provided the tracks along which the GA elements were transported to the cell centre. Once present in close proximity to each other, these large elements tangentially connected to form a single membrane unit in a Golgi-based MT-dependent manner. These results indicate that centrosome- and Golgi-derived MTs have different roles in the Golgi assembly process: centrosomal MTs ensure the pericentrosomal location of the GA, whereas Golgi-nucleated MTs are responsible for Golgi ribbon integrity. This mechanism is surely operating in Golgi re-assembly that occurs every time a cell exits mitosis [73,74] (see §3*d*). However, the scenario in mitosis appears to be more complicated. In many cell types, GA reassembly during cytokinesis occurs at two different subcellular locations: one Golgi ribbon is formed around the centrosome, and the other one is situated next to the midbody and flanks the intracellular bridge between the two daughter cells. These two ribbons then coalesce at the cell centre and eventually form a single unit. To understand how the complex and evolving geometry of MT network during telophase and cytokinesis contributes to the formation of the Golgi ribbon at the mitotic exit deserves careful analysis in future studies.

Fusion of Golgi stacks into a polarized Golgi ribbon is a complex process that implies lateral linking, and subsequent fusion, of homotypic cisternae. It requires not only proper orientation of polarized stacks but also precise recognition of cisternal identity. Golgi-nucleated MTs that grow tangential to stacks might contribute to this process by allowing Golgi stacks to align properly, thus facilitating successive linking and fusion events. Interestingly, the *cis*-Golgi protein GRASP65 found in a stable complex with GM130 [75] plays an important role in the lateral linking of the *cis*-cisternae by forming anti-parallel homo-oligomers in *trans* [76]. These oligomers bring cisternae into close contact, thereby allowing membrane fusion to proceed. Based on that, the hypothesis that GM130 connects MT-nucleation and lateral membrane linking machineries at the *cis*-face of the GA is appealing. This would certainly facilitate the elaborate process of fusing hundreds of Golgi stacks into a single highly polarized structure.

Several studies have emphasized the intrinsic asymmetric nature of Golgi-derived MT arrays [5,66,73]. This asymmetry is relevant for polarized cell organization that, in turn, is essential for cell migration. Cells lacking Golgi-derived MTs migrate more slowly in wound-healing assays in spite of proper pericentrosomal positionioning of the GA and coordinated reorientation of both the centrosome and the GA towards the leading edge [36,66]. This clearly identifies Golgi-nucleated MTs as important players in regulating directional migration, probably by establishing preferential secretion paths towards the leading edge in migrating cells. Consistently, directional but not general secretion is affected under these conditions. It should be noted that disrupting Golgi–centrosome association has a stronger negative effect on cell polarity and migration than simply inhibiting MT nucleation [36]. As a matter of fact, dislocation of the polarity axis induced by the expression of the N-terminus of AKAP450 results not only in a reduced migration rate but also in an aberrant migration pattern with cells moving in the wrong direction [36]. This is in agreement with previous evidence that motile cells require a polarized Golgi complex in proximity to the centrosome for proper directional post-Golgi trafficking and directional cell migration [77].

In conclusion, coupling of centrosome- and Golgi-derived MT activities ensures the correct formation and location of the Golgi ribbon, which is vital for cellular functions that require polarized secretion and directional migration.

### (d) Mitotic regulation of Golgi-associated microtubule nucleation

During cell division, the single Golgi ribbon must be divided into the two daughter cells. To prepare for proper segregation, the ribbon first unlinks into individual stacks, which further undergo unstacking and vesiculation. These mitotic Golgi membranes are then partitioned between the two daughter cells where they reassemble in a single Golgi ribbon after cytokinesis [78]. Disassembly of the Golgi complex during mitosis is not a conserved phenomenon among eukaryotes, suggesting that it is not strictly required to ensure Golgi inheritance. Why vertebrate cells have developed such a sophisticated strategy is not yet fully understood. It has been proposed that Golgi stack unlinking in late G2 is a necessary step for cells to enter into mitosis rather than only a passive consequence of mitosis. As a matter of fact, experimentally induced block of Golgi unlinking in G2 delays entry into mitosis [79]. Since Golgi-nucleated MTs play a critical role in the fusion of Golgi stacks into a single unit, one can safely assume that the machinery controlling this process is subjected to mitotic regulation. And indeed, this is what has recently been demonstrated in a study by Maia *et al.* [74] addressing the question of the ability of Golgi membranes to nucleate MTs during the cell cycle. The MT nucleation capacity of Golgi membranes remains unchanged until late prophase, when fragmented Golgi membranes start to be visible, then it is strongly downregulated during metaphase and regained in telophase.

Upon mitotic exit, Golgi stacks are re-formed by tightly regulated fusion of small Golgi membrane vesicles into cisternae that undergo subsequent stacking. Formation of stacks is MT-independent and can be reconstituted in a cell-free system or in MT-devoid cells [78]. However, reassembly into a functional Golgi ribbon in late mitosis was shown to be dependent on MTs. How this process is regulated at the molecular level is far from clear. The aforementioned Maia *et al.* study also analysed the reformation of the Golgi ribbon at mitotic exit. In the presence of a dominant negative mutant of AKAP450, reforming Golgi stacks accumulate at the cell centre during telophase but they remain small and fragmented [74]. Therefore, Golgi-associated AKAP450-dependent MT nucleation is essential to bring together emerging Golgi stacks in the course of post-mitotic assembly, thus promoting Golgi stack fusion and efficient Golgi ribbon formation. In line with that, a striking observation was reported by Wei & Seemann [80]. They observed that, while reformation of Golgi stacks is independent of the mitotic spindle, the fusion of stacks into a ribbon requires factors that segregate with the mitotic spindle. Since AKAP450, CDK5Rap2, myomegalin and CLASPs are components of the mitotic spindle, it is tempting to speculate that a pool of these proteins is released from the spindle during telophase and somehow participates in post-mitotic Golgi reassembly.

### (e) Golgi nucleation in differentiated cells

Terminal differentiation of mammalian cells involves extensive changes in cell morphology and in subcellular architecture. The best-studied examples include epithelial, muscle and neuronal cells. A common feature of these cell types is that MTs are reorganized during differentiation into non-centrosomal complex arrays by mechanisms that are not yet well understood. Recently available data demostrate that Golgi-nucleated MTs play a critical role in MT reorganization taking place during both muscle and neuron differentiation [8,65].

Reorganization of cell architecture during skeletal muscle differentiation implicates complete and simultaneous remodelling of the centrosome, the MT network and the secretory system. Such reorganization has been mainly studied in myoblasts induced to differentiate into multinucleated myotubes *in vitro*. Myoblasts exhibit a classical MT network focused on juxtanuclear centrosomes surrounded by the GA, whereas myotubes possess numerous MTs organized in parallel, without any apparent nucleation centres. First insights into the mechanisms underlying such a transition came from pioneering studies from Bornens’ group [81,82]. They showed that during myotube differentiation both the PCM and the GA redistributed to the nuclear envelope where they formed perinuclear belts. Concomitantly, nuclei acquired MT nucleation capacity. Perinuclear belts were then shown to contain pericentrin, γ-tubulin and ninein that could account for the MT nucleating activity of myotube nuclei. Interestingly, in short-time MT regrowth experiments, new MTs not only formed at the perinuclear ring but also appeared as asters growing from cytoplasmic nucleating sites. More than 85% of these MT regrowth foci were identified as Golgi elements that in fused myotubes not only surrounded nuclei but also extended between them [83–85].

Muscle tissue cultures have been useful for studying the first phase of differentiation of myoblasts into multinucleated myotubes. However, cultured myotubes do not mature into the fibres that form muscle. Muscle fibres are flat cylinders with actomyosin filaments occupying the cytoplasm almost completely. Nuclei, organelles and MTs concentrate in a thin layer of cytoplasm between filaments and the plasma membrane. These MTs form a grid-like network with very few clear starting and end points. Remarkably, Golgi elements are positioned at the vertices of the MT lattice in a unique organization. Using confocal, intravital and super-resolution microscopy, Ralston's group nicely demonstrated that muscle MTs are dynamic and form small bundles which build a stable MT network [8]. They found that static Golgi elements, located at MT intersections of the orthogonal grid of MTs, were the major sites of muscle MT nucleation in addition to the nuclear envelope, generating in this way an unusual MT network. They detected γ-tubulin and pericentrin on Golgi elements of muscle fibres but not AKAP450 or CLASP2. However, since multiple AKAP450 isoforms exist and not all anti-AKAP450 antibodies are able to recognize Golgi-associated AKAP450 fraction [30], the presence of AKAP450 at the surface of Golgi elements cannot be formally excluded. Nevertheless, pericentrin and AKAP450 are structurally and functionally related proteins, suggesting that a similar mechanism to that found in cultured cells could also regulate MT nucleation at the Golgi in muscles.

Neurons are some of the most complex and highly polarized cells in animals. They differentiate from round cells that gradually acquire polarity, to a multipolar stage with many neurites and finally to the formation of one single axon and multiple dendrites. MT nucleation is essential for proper formation and maintenance of both dendritic and axonal branches [86]. How the polarized MT array in neurons is generated is still an important open question. It has been proposed that MTs nucleated at the centrosomes are cleaved and then transported to the proper compartment. Alternatively, MTs might be severed in the periphery and serve as scaffolds for nucleation/polymerization [86]. Recent studies have shown, however, that the centrosome loses its function as the major MT-organizing centre during neuronal development and that, in fact, acentrosomal nucleation occurs in neurons [7,87]. In neurons, the GA comprises Golgi stacks located within the soma and Golgi elements termed Golgi outposts present along the dendrites, at dendritic branch points and at the distal tips. Using time-lapse microscopy and *in vitro* experiments, Ori-McKenney *et al.* [65] have investigated the origin of MTs within the dendritic arbour of a specific type of neurons in *Drosophila*. They found that Golgi outposts can directly nucleate MTs through the dendritic arbour. This acentrosomal MT nucleation requires γ-tubulin and the *Drosophila* homologue of AKAP450. Partially purified Golgi outposts containing both proteins were able to nucleate MTs in *in vitro* assays. Most importantly, they showed that Golgi outpost-associated MT nucleation regulates distal dendritic branching and is critical for terminal branch stabilization. It is worth mentioning that Golgi outposts are absent in the axon, which is a long primary branch with uniform MT polarity. By contrast, the dendritic arbour is an intricate array of branches, where MT polarity depends on the branch length. In neurons lacking cytoplasmic dynein, Golgi outposts mislocalize to the axon which appears branched and contains MTs of mixed polarity [88]. Interestingly, small MT bundles growing from Golgi elements in muscle fibres also contain MTs of mixed polarity. Therefore, it can be speculated that generation of MT arrays with mixed MT orientation might be a property of Golgi-associated MT nucleation in complex morphogenetic processes.

## 4. Concluding remarks and perspectives

A comparison between the MT nucleation process at the centrosome and at the GA highlights some common features and interesting differences. Indeed, data presented in this review show that the GA uses classical centrosomal proteins for its MT nucleation activity. These data further suggest that centrosome-associated proteins can function fully independently of the centrosome. While this assumption is still valid, the network-like distribution of proteins such as AKAP450 or CDK5Rap2 extending from the centrosome towards the GA, suggests the existence of direct connections between these two organelles (see figure 2 for a working model). Admittedly, this connection would facilitate the well-known coordinated behaviour of both organelles in physiological processes that require MTs to be dynamic, such as cell migration, Golgi reassembly after mitosis, and the formation of the immunological synapse [49]. Additionally, the existence of PCM protein networks connecting the centrosome and the GA raises the interesting question of how precisely to define the limit of the centrosome in mammalian cells. *In vivo* analysis and super high-resolution imaging techniques will certainly help to refine our knowledge of the organization of this crucial subcellular region. Despite the recent steps forward in our understanding of the MT nucleation process at the GA, a coherent view about how Golgi-associated PCM proteins interact with each other and with CLASPs in order to orchestrate this process is still lacking. The mechanism by which Golgi-based MTs cooperate with membrane tethering and fusion machineries to generate a single membrane unit also remains unknown. Thus, integrative studies will be useful to assemble in common networks proteins involved in controlling MT formation and those regulating Golgi ribbon assembly and membrane trafficking.

The major differences between centrosome-nucleated and Golgi-nucleated MTs stem from their geometry and nature. It has been known for long time that MTs colocalizing with the GA are highly enriched in post-translationally modified tubulins, in particular detyrosinated and acetylated α-tubulin [54]. In this regard, the most obvious questions are how and why the molecular machinery responsible for such modifications specifically targets the Golgi subpopulation of MTs.

Data examined in this review also reveal an important role of the GA in organizing complex and specialized MT arrays that carry out specific functions in differentiated cells. Hopefully, the recent discoveries in muscles and neurons will be soon extended to other cell types with equally complex MT arrays. Particularly relevant will be a thorough understanding of how MT nucleation at the GA contributes to MT remodelling during the establishment of apico-basal polarity in epithelial cells.

Finally, since MT nucleation at the centrosome and the GA is probably differently, yet coordinatively regulated in a cell cycle- and cell type-dependent manner, deciphering the signalling pathways underlying such regulation will no doubt deserve more attention in years to come. Further efforts should also be made to understand this regulation better in different biological contexts, for example, during animal development and disease pathogenesis.

## References

[1] Kollman JM Merdes A Mourey L Agard DA 2011 Microtubule nucleation by gamma-tubulin complexes. Nat. Rev. Mol. Cell Biol. 12, 709–721. (10.1038/nrm3209) 21993292 PMC7183383

[2] Moudjou M Bordes N Paintrand M Bornens M 1996 gamma-Tubulin in mammalian cells: the centrosomal and the cytosolic forms. J. Cell Sci. 109, 875–887. 8718679 10.1242/jcs.109.4.875

[3] Bornens M 2002 Centrosome composition and microtubule anchoring mechanisms. Curr. Opin. Cell Biol. 14, 25–34. (10.1016/S0955-0674(01)00290-3) 11792541

[4] Teixido-Travesa N Roig J Luders J 2012 The where, when and how of microtubule nucleation: one ring to rule them all. J. Cell Sci. 125, 4445–4456. (10.1242/jcs.106971) 23132930

[5] Efimov A 2007 Asymmetric CLASP-dependent nucleation of noncentrosomal microtubules at the trans-Golgi network. Dev. Cell 12, 917–930. (10.1016/j.devcel.2007.04.002) 17543864 PMC2705290

[6] Khodjakov A Rieder CL 1999 The sudden recruitment of gamma-tubulin to the centrosome at the onset of mitosis and its dynamic exchange throughout the cell cycle, do not require microtubules. J. Cell Biol. 146, 585–596. (10.1083/jcb.146.3.585) 10444067 PMC2150561

[7] Stiess M Maghelli N Kapitein LC Gomis-Ruth S Wilsch-Brauninger M Hoogenraad CC Tolic-Norrelykke IM Bradke F 2010 Axon extension occurs independently of centrosomal microtubule nucleation. Science 327, 704–707. (10.1126/science.1182179) 20056854

[8] Oddoux S Zaal KJ Tate V Kenea A Nandkeolyar SA Reid E Liu W Ralston E 2013 Microtubules that form the stationary lattice of muscle fibers are dynamic and nucleated at Golgi elements. J. Cell Biol. 203, 205–213. (10.1083/jcb.201304063) 24145165 PMC3812964

[9] Mennella V Keszthelyi B McDonald KL Chhun B Kan F Rogers GC Huang B Agard DA 2012 Subdiffraction-resolution fluorescence microscopy reveals a domain of the centrosome critical for pericentriolar material organization. Nat. Cell Biol. 14, 1159–1168. (10.1038/ncb2597) 23086239 PMC3767400

[10] Sonnen KF Schermelleh L Leonhardt H Nigg EA 2012 3D-structured illumination microscopy provides novel insight into architecture of human centrosomes. Biol. Open 1, 965–976. (10.1242/bio.20122337) 23213374 PMC3507176

[11] Lawo S Hasegan M Gupta GD Pelletier L 2012 Subdiffraction imaging of centrosomes reveals higher-order organizational features of pericentriolar material. Nat. Cell Biol. 14, 1148–1158. (10.1038/ncb2591) 23086237

[12] Fu J Glover DM 2012 Structured illumination of the interface between centriole and peri-centriolar material. Open Biol. 2, 120104 (10.1098/rsob.120104) 22977736 PMC3438536

[13] Hutchins JR 2010 Systematic analysis of human protein complexes identifies chromosome segregation proteins. Science 328, 593–599. (10.1126/science.1181348) 20360068 PMC2989461

[14] Teixido-Travesa N Villen J Lacasa C Bertran MT Archinti M Gygi SP Caelles C Roig J Luders J 2010 The gammaTuRC revisited: a comparative analysis of interphase and mitotic human gammaTuRC redefines the set of core components and identifies the novel subunit GCP8. Mol. Biol. Cell 21, 3963–3972. (10.1091/mbc.E10-05-0408) 20861304 PMC2982109

[15] Luders J Patel UK Stearns T 2006 GCP-WD is a gamma-tubulin targeting factor required for centrosomal and chromatin-mediated microtubule nucleation. Nat. Cell Biol. 8, 137–147. (10.1038/ncb1349) 16378099

[16] Kollman JM Polka JK Zelter A Davis TN Agard DA 2010 Microtubule nucleating gamma-TuSC assembles structures with 13-fold microtubule-like symmetry. Nature 466, 879–882. (10.1038/nature09207) 20631709 PMC2921000

[17] Manning JA Shalini S Risk JM Day CL Kumar S 2010 A direct interaction with NEDD1 regulates gamma-tubulin recruitment to the centrosome. PLoS ONE 5, e9618 (10.1371/journal.pone.0009618) 20224777 PMC2835750

[18] Zhu F 2008 The mammalian SPD-2 ortholog Cep192 regulates centrosome biogenesis. Curr. Biol. 18, 136–141. (10.1016/j.cub.2007.12.055) 18207742

[19] Gomez-Ferreria MA Bashkurov M Helbig AO Larsen B Pawson T Gingras AC Pelletier L 2012 Novel NEDD1 phosphorylation sites regulate gamma-tubulin binding and mitotic spindle assembly. J. Cell Sci. 125, 3745–3751. (10.1242/jcs.105130) 22595525

[20] Haren L Stearns T Luders J 2009 Plk1-dependent recruitment of gamma-tubulin complexes to mitotic centrosomes involves multiple PCM components. PLoS ONE 4, e5976 (10.1371/journal.pone.0005976) 19543530 PMC2695007

[21] Casenghi M Meraldi P Weinhart U Duncan PI Korner R Nigg EA 2003 Polo-like kinase 1 regulates Nlp, a centrosome protein involved in microtubule nucleation. Dev. Cell 5, 113–125. (10.1016/S1534-5807(03)00193-X) 12852856

[22] Casenghi M Barr FA Nigg EA 2005 Phosphorylation of Nlp by Plk1 negatively regulates its dynein–dynactin-dependent targeting to the centrosome. J. Cell Sci. 118, 5101–5108. (10.1242/jcs.02622) 16254247

[23] Flory MR Davis TN 2003 The centrosomal proteins pericentrin and kendrin are encoded by alternatively spliced products of one gene. Genomics 82, 401–405. (10.1016/S0888-7543(03)00119-8) 12906865

[24] Zimmerman WC Sillibourne J Rosa J Doxsey SJ 2004 Mitosis-specific anchoring of gamma tubulin complexes by pericentrin controls spindle organization and mitotic entry. Mol. Biol. Cell 15, 3642–3657. (10.1091/mbc.E03-11-0796) 15146056 PMC491825

[25] Takahashi M Yamagiwa A Nishimura T Mukai H Ono Y 2002 Centrosomal proteins CG-NAP and kendrin provide microtubule nucleation sites by anchoring gamma-tubulin ring complex. Mol. Biol. Cell 13, 3235–3245. (10.1091/mbc.E02-02-0112) 12221128 PMC124155

[26] Gillingham AK Munro S 2000 The PACT domain, a conserved centrosomal targeting motif in the coiled-coil proteins AKAP450 and pericentrin. EMBO Rep. 1, 524–529. (10.1093/embo-reports/kvd105) 11263498 PMC1083777

[27] Fong KW Choi YK Rattner JB Qi RZ 2008 CDK5RAP2 is a pericentriolar protein that functions in centrosomal attachment of the gamma-tubulin ring complex. Mol. Biol. Cell 19, 115–125. (10.1091/mbc.E07-04-0371) 17959831 PMC2174194

[28] Choi YK Liu P Sze SK Dai C Qi RZ 2010 CDK5RAP2 stimulates microtubule nucleation by the gamma-tubulin ring complex. J. Cell Biol. 191, 1089–1095. (10.1083/jcb.201007030) 21135143 PMC3002024

[29] Roubin R Acquaviva C Chevrier V Sedjai F Zyss D Birnbaum D Rosnet O 2013 Myomegalin is necessary for the formation of centrosomal and Golgi-derived microtubules. Biol. Open 2, 238–250. (10.1242/bio.20123392) 23430395 PMC3575658

[30] Keryer G Witczak O Delouvee A Kemmner WA Rouillard D Tasken K Bornens M 2003 Dissociating the centrosomal matrix protein AKAP450 from centrioles impairs centriole duplication and cell cycle progression. Mol. Biol. Cell 14, 2436–2446. (10.1091/mbc.E02-09-0614) 12808041 PMC194891

[31] Lee K Rhee K 2011 PLK1 phosphorylation of pericentrin initiates centrosome maturation at the onset of mitosis. J. Cell Biol. 195, 1093–1101. (10.1083/jcb.201106093) 22184200 PMC3246884

[32] Buchman JJ Tseng HC Zhou Y Frank CL Xie Z Tsai LH 2010 Cdk5rap2 interacts with pericentrin to maintain the neural progenitor pool in the developing neocortex. Neuron 66, 386–402. (10.1016/j.neuron.2010.03.036) 20471352

[33] Barr AR Kilmartin JV Gergely F 2010 CDK5RAP2 functions in centrosome to spindle pole attachment and DNA damage response. J. Cell Biol. 189, 23–39. (10.1083/jcb.200912163) 20368616 PMC2854379

[34] Delgehyr N Sillibourne J Bornens M 2005 Microtubule nucleation and anchoring at the centrosome are independent processes linked by ninein function. J. Cell Sci. 118, 1565–1575. (10.1242/jcs.02302) 15784680

[35] Kim HS Takahashi M Matsuo K Ono Y 2007 Recruitment of CG-NAP to the Golgi apparatus through interaction with dynein–dynactin complex. Genes Cells 12, 421–434. (10.1111/j.1365-2443.2007.01055.x) 17352745

[36] Hurtado L Caballero C Gavilan MP Cardenas J Bornens M Rios RM 2011 Disconnecting the Golgi ribbon from the centrosome prevents directional cell migration and ciliogenesis. J. Cell Biol. 193, 917–933. (10.1083/jcb.201011014) 21606206 PMC3105543

[37] Yan X Habedanck R Nigg EA 2006 A complex of two centrosomal proteins, CAP350 and FOP, cooperates with EB1 in microtubule anchoring. Mol. Biol. Cell 17, 634–644. (10.1091/mbc.E05-08-0810) 16314388 PMC1356575

[38] Takahashi M Shibata H Shimakawa M Miyamoto M Mukai H Ono Y 1999 Characterization of a novel giant scaffolding protein, CG-NAP, that anchors multiple signaling enzymes to centrosome and the Golgi apparatus. J. Biol. Chem. 274, 17 267–17 274. (10.1074/jbc.274.24.17267) 10358086

[39] Wang Z Wu T Shi L Zhang L Zheng W Qu JY Niu R Qi RZ 2010 Conserved motif of CDK5RAP2 mediates its localization to centrosomes and the Golgi complex. J. Biol. Chem. 285, 22 658–22 665. (10.1074/jbc.M110.105965) PMC2903348 20466722

[40] Verde I Pahlke G Salanova M Zhang G Wang S Coletti D Onuffer J Jin SL Conti M 2001 Myomegalin is a novel protein of the Golgi/centrosome that interacts with a cyclic nucleotide phosphodiesterase. J. Biol. Chem. 276, 11 189–11 198. (10.1074/jbc.M006546200) 11134006

[41] Hoppeler-Lebel A Celati C Bellett G Mogensen MM Klein-Hitpass L Bornens M Tassin AM 2007 Centrosomal CAP350 protein stabilises microtubules associated with the Golgi complex. J. Cell Sci. 120, 3299–3308. (10.1242/jcs.013102) 17878239

[42] Preuss D Mulholland J Franzusoff A Segev N Botstein D 1992 Characterization of the *Saccharomyces* Golgi complex through the cell cycle by immunoelectron microscopy. Mol. Biol. Cell 3, 789–803. (10.1091/mbc.3.7.789) 1381247 PMC275635

[43] Henderson GP Gan L Jensen GJ 2007 3-D ultrastructure of *O. tauri*: electron cryotomography of an entire eukaryotic cell. PLoS ONE 2, e749 (10.1371/journal.pone.0000749) 17710148 PMC1939878

[44] He CY Ho HH Malsam J Chalouni C West CM Ullu E Toomre D Warren G 2004 Golgi duplication in *Trypanosoma brucei*. J. Cell Biol. 165, 313–321. (10.1083/jcb.200311076) 15138289 PMC2172185

[45] Mogelsvang S Gomez-Ospina N Soderholm J Glick BS Staehelin LA 2003 Tomographic evidence for continuous turnover of Golgi cisternae in *Pichia pastoris*. Mol. Biol. Cell 14, 2277–2291. (10.1091/mbc.E02-10-0697) 12808029 PMC260745

[46] daSilva LL Snapp EL Denecke J Lippincott-Schwartz J Hawes C Brandizzi F 2004 Endoplasmic reticulum export sites and Golgi bodies behave as single mobile secretory units in plant cells. Plant Cell 16, 1753–1771. (10.1105/tpc.022673) 15208385 PMC514159

[47] Kondylis V Rabouille C 2009 The Golgi apparatus: lessons from *Drosophila*. FEBS Lett. 583, 3827–3838. (10.1016/j.febslet.2009.09.048) 19800333

[48] Rios RM Bornens M 2003 The Golgi apparatus at the cell centre. Curr. Opin. Cell Biol. 15, 60–66. (10.1016/S0955-0674(02)00013-3) 12517705

[49] Yadav S Linstedt AD 2011 Golgi positioning. Cold Spring Harbor Perspect. Biol. 3, a000521 (10.1101/cshperspect.a005322) PMC3101843 21504874

[50] Presley JF Cole NB Schroer TA Hirschberg K Zaal KJ Lippincott-Schwartz J 1997 ER-to-Golgi transport visualized in living cells. Nature 389, 81–85. (10.1038/38891) 9288971

[51] Corthesy-Theulaz I Pauloin A Pfeffer SR 1992 Cytoplasmic dynein participates in the centrosomal localization of the Golgi complex. J. Cell Biol. 118, 1333–1345. (10.1083/jcb.118.6.1333) 1387874 PMC2289611

[52] Harada A Takei Y Kanai Y Tanaka Y Nonaka S Hirokawa N 1998 Golgi vesiculation and lysosome dispersion in cells lacking cytoplasmic dynein. J. Cell Biol. 141, 51–59. (10.1083/jcb.141.1.51) 9531547 PMC2132725

[53] Cole NB Sciaky N Marotta A Song J Lippincott-Schwartz J 1996 Golgi dispersal during microtubule disruption: regeneration of Golgi stacks at peripheral endoplasmic reticulum exit sites. Mol. Biol. Cell 7, 631–650. (10.1091/mbc.7.4.631) 8730104 PMC275914

[54] Thyberg J Moskalewski S 1999 Role of microtubules in the organization of the Golgi complex. Exp. Cell Res. 246, 263–279. (10.1006/excr.1998.4326) 9925741

[55] Yadav S Puthenveedu MA Linstedt AD 2012 Golgin160 recruits the dynein motor to position the Golgi apparatus. Dev. Cell 23, 153–165. (10.1016/j.devcel.2012.05.023) 22814606 PMC3417773

[56] Yadav S Puri S Linstedt AD 2009 A primary role for Golgi positioning in directed secretion, cell polarity, wound healing. Mol. Biol. Cell 20, 1728–1736. (10.1091/mbc.E08-10-1077) 19158377 PMC2655245

[57] Rios RM Sanchis A Tassin AM Fedriani C Bornens M 2004 GMAP-210 recruits gamma-tubulin complexes to *cis*-Golgi membranes and is required for Golgi ribbon formation. Cell 118, 323–335. (10.1016/j.cell.2004.07.012) 15294158

[58] Infante C Ramos-Morales F Fedriani C Bornens M Rios RM 1999 GMAP-210, A *cis*-Golgi network-associated protein, is a minus end microtubule-binding protein. J. Cell Biol. 145, 83–98. (10.1083/jcb.145.1.83) 10189370 PMC2148210

[59] Drin G Morello V Casella JF Gounon P Antonny B 2008 Asymmetric tethering of flat and curved lipid membranes by a golgin. Science 320, 670–673. (10.1126/science.1155821) 18451304

[60] Cardenas J Rivero S Goud B Bornens M Rios RM 2009 Golgi localisation of GMAP210 requires two distinct *cis*-membrane binding mechanisms. BMC Biol. 7, 56 (10.1186/1741-7007-7-56) 19715559 PMC2744908

[61] Smits P 2010 Lethal skeletal dysplasia in mice and humans lacking the golgin GMAP-210. New Engl. J. Med. 362, 206–216. (10.1056/NEJMoa0900158) 20089971 PMC3108191

[62] Follit JA San Agustin JT Xu F Jonassen JA Samtani R Lo CW Pazour GJ 2008 The golgin GMAP210/TRIP11 anchors IFT20 to the Golgi complex. PLoS Genet. 4, e1000315 (10.1371/journal.pgen.1000315) 19112494 PMC2602600

[63] Broekhuis JR Rademakers S Burghoorn J Jansen G 2013 SQL-1, homologue of the Golgi protein GMAP210, modulates intraflagellar transport in *C. elegans*. J. Cell Sci. 126, 1785–1795. (10.1242/jcs.116640) 23444385

[64] Chabin-Brion K Marceiller J Perez F Settegrana C Drechou A Durand G Pous C 2001 The Golgi complex is a microtubule-organizing organelle. Mol. Biol. Cell 12, 2047–2060. (10.1091/mbc.12.7.2047) 11452002 PMC55652

[65] Ori-McKenney KM Jan LY Jan YN 2012 Golgi outposts shape dendrite morphology by functioning as sites of acentrosomal microtubule nucleation in neurons. Neuron 76, 921–930. (10.1016/j.neuron.2012.10.008) 23217741 PMC3523279

[66] Rivero S Cardenas J Bornens M Rios RM 2009 Microtubule nucleation at the *cis*-side of the Golgi apparatus requires AKAP450 and GM130. EMBO J. 28, 1016–1028. (10.1038/emboj.2009.47) 19242490 PMC2683699

[67] Sawin KE Lourenco PC Snaith HA 2004 Microtubule nucleation at non-spindle pole body microtubule-organizing centers requires fission yeast centrosomin-related protein mod20p. Curr. Biol. 14, 763–775. (10.1016/j.cub.2004.03.042) 15120067

[68] Kawaguchi S Zheng Y 2004 Characterization of a *Drosophila* centrosome protein CP309 that shares homology with kendrin and CG-NAP. Mol. Biol. Cell 15, 37–45. (10.1091/mbc.E03-03-0191) 14565985 PMC307525

[69] Zhu X Kaverina I 2013 Golgi as an MTOC: making microtubules for its own good. Histochem. Cell Biol. 140, 361–367. (10.1007/s00418-013-1119-4) 23821162 PMC3748218

[70] Moreno-Mateos MA Espina AG Torres B Gamez del Estal MM Romero-Franco A Rios RM Pintor-Toro JA 2011 PTTG1/securin modulates microtubule nucleation and cell migration. Mol. Biol. Cell 22, 4302–4311. (10.1091/mbc.E10-10-0838) 21937724 PMC3216656

[71] Fong KW Hau SY Kho YS Jia Y He L Qi RZ 2009 Interaction of CDK5RAP2 with EB1 to track growing microtubule tips and to regulate microtubule dynamics. Mol. Biol. Cell 20, 3660–3670. (10.1091/mbc.E09-01-0009) 19553473 PMC2777926

[72] Marsh BJ Mastronarde DN Buttle KF Howell KE McIntosh JR 2001 Organellar relationships in the Golgi region of the pancreatic beta cell line, HIT-T15, visualized by high resolution electron tomography. Proc. Natl Acad. Sci. USA 98, 2399–2406. (10.1073/pnas.051631998) 11226251 PMC30150

[73] Miller PM Folkmann AW Maia AR Efimova N Efimov A Kaverina I 2009 Golgi-derived CLASP-dependent microtubules control Golgi organization and polarized trafficking in motile cells. Nat. Cell Biol. 11, 1069–1080. (10.1038/ncb1920) 19701196 PMC2748871

[74] Maia AR Zhu X Miller P Gu G Maiato H Kaverina I 2013 Modulation of Golgi-associated microtubule nucleation throughout the cell cycle. Cytoskeleton (Hoboken) 70, 32–43. (10.1002/cm.21079) 23027431 PMC3574797

[75] Puthenveedu MA Bachert C Puri S Lanni F Linstedt AD 2006 GM130 and GRASP65-dependent lateral cisternal fusion allows uniform Golgi-enzyme distribution. Nat. Cell Biol. 8, 238–248. (10.1038/ncb1366) 16489344

[76] Sengupta D Truschel S Bachert C Linstedt AD 2009 Organelle tethering by a homotypic PDZ interaction underlies formation of the Golgi membrane network. J. Cell Biol. 186, 41–55. (10.1083/jcb.200902110) 19581411 PMC2712994

[77] Millarte V Farhan H 2012 The Golgi in cell migration: regulation by signal transduction and its implications for cancer cell metastasis. Sci. World J. 2012, 498278 (10.1100/2012/498278) PMC3353474 22623902

[78] Wei JH Seemann J 2010 Unraveling the Golgi ribbon. Traffic 11, 1391–1400. (10.1111/j.1600-0854.2010.01114.x) 21040294 PMC4221251

[79] Sutterlin C Hsu P Mallabiabarrena A Malhotra V 2002 Fragmentation and dispersal of the pericentriolar Golgi complex is required for entry into mitosis in mammalian cells. Cell 109, 359–369. (10.1016/S0092-8674(02)00720-1) 12015985

[80] Wei JH Seemann J 2009 The mitotic spindle mediates inheritance of the Golgi ribbon structure. J. Cell Biol. 184, 391–397. (10.1083/jcb.200809090) 19188490 PMC2646559

[81] Tassin AM Maro B Bornens M 1985 Fate of microtubule-organizing centers during myogenesis *in vitro*. J. Cell Biol. 100, 35–46. (10.1083/jcb.100.1.35) 3880758 PMC2113478

[82] Tassin AM Paintrand M Berger EG Bornens M 1985 The Golgi apparatus remains associated with microtubule organizing centers during myogenesis. J. Cell Biol. 101, 630–638. (10.1083/jcb.101.2.630) 3894380 PMC2113672

[83] Bugnard E Zaal KJ Ralston E 2005 Reorganization of microtubule nucleation during muscle differentiation. Cell Motil. Cytoskeleton 60, 1–13. (10.1002/cm.20042) 15532031

[84] Lu Z Joseph D Bugnard E Zaal KJ Ralston E 2001 Golgi complex reorganization during muscle differentiation: visualization in living cells and mechanism. Mol. Biol. Cell 12, 795–808. (10.1091/mbc.12.4.795) 11294887 PMC32267

[85] Zaal KJ Reid E Mousavi K Zhang T Mehta A Bugnard E Sartorelli V Ralston E 2011 Who needs microtubules? Myogenic reorganization of MTOC, Golgi complex and ER exit sites persists despite lack of normal microtubule tracks. PLoS ONE 6, e29057 (10.1371/journal.pone.0029057) 22216166 PMC3246457

[86] Kuijpers M Hoogenraad CC 2011 Centrosomes, microtubules and neuronal development. Mol. Cell. Neurosci. 48, 349–358. (10.1016/j.mcn.2011.05.004) 21722732

[87] Nguyen MM Stone MC Rolls MM 2011 Microtubules are organized independently of the centrosome in *Drosophila* neurons. Neural Dev. 6, 38 (10.1186/1749-8104-6-38) 22145670 PMC3271965

[88] Zheng Y Wildonger J Ye B Zhang Y Kita A Younger SH Zimmerman S Jan LY Jan YN 2008 Dynein is required for polarized dendritic transport and uniform microtubule orientation in axons. Nat. Cell Biol. 10, 1172–1180. (10.1038/ncb1777) 18758451 PMC2588425

